# Early-Onset Type 2 Diabetes: A Comprehensive Review From Adolescence to Adulthood

**DOI:** 10.7759/cureus.98896

**Published:** 2025-12-10

**Authors:** Nasser A Alomari, Mohammed S Alghamdi, Ghadah A Algarni, Mutaz A Ezzatallah, Rawan A Barakat, Amer Edris, Faisal Alfageeh, Zeyad H Althumali, Aseel S Felemban

**Affiliations:** 1 Family Medicine, King Fahad Armed Forces Hospital, Jeddah, SAU; 2 Internal Medicine, King Fahad Armed Forces Hospital, Jeddah, SAU; 3 Pulmonology, King Fahad Armed Forces Hospital, Jeddah, SAU; 4 Neurosurgery, King Abdullah Medical Complex, Jeddah, SAU; 5 General Practice, King Abdulaziz University, Jeddah, SAU; 6 General Practice, King Abdulaziz University Hospital, Jeddah, SAU; 7 Medicine, Faculty of Medicine, King Abdulaziz University, Jeddah, SAU; 8 Family Medicine, King Fahad General Hospital, Al-Baha, SAU; 9 Preventive Medicine, King Abdulaziz Medical City, Jeddah, SAU; 10 General Practice, Ibn Sina National College, Jeddah, SAU

**Keywords:** adolescents, early-onset diabetes, insulin resistance, obesity, β-cell dysfunction

## Abstract

Type 2 diabetes mellitus, traditionally considered a disease of adults, is increasingly being diagnosed in younger individuals, including adolescents and young adults. This review provides a comprehensive examination of early-onset type 2 diabetes, focusing on its unique characteristics, underlying mechanisms, and clinical implications. The condition develops through interactions between genetic susceptibility, lifestyle factors, and metabolic changes during growth and development. Youth diagnosed with type 2 diabetes face the challenge of managing a chronic disease over many decades of life. Effective management requires a multifaceted approach that addresses both medical and psychosocial needs, incorporating lifestyle modifications, appropriate pharmacological interventions, and coordinated healthcare delivery. The transition from pediatric to adult care represents a critical period requiring careful attention to ensure continuity of treatment. Early-onset type 2 diabetes represents a pressing public health challenge that demands a shift in focus from treatment to prevention, emphasizing early screening of at-risk youth, promoting healthy environments, and incorporating family-centered interventions to reduce the burden of this condition on individuals and healthcare systems.

## Introduction and background

Diabetes mellitus (DM) is a global epidemic and a major cause of premature death. It affects one in 10 individuals aged 20 to 79 years, and diagnosis rates continue to rise worldwide [1]. The age at diagnosis determines whether diabetes is classified as adult-onset or early-onset. Type 2 DM (T2DM) diagnosed after age 50 is termed "adult-onset diabetes," while T2DM diagnosed before age 40 is classified as early-onset T2DM [2,3]. The increase in early-onset T2DM cases is particularly concerning because individuals with early-onset disease face greater risks of complications and mortality than those diagnosed later in life [4]. The incidence of DM has increased from 56.02 per 100,000 in 1990 to 123.86 per 100,000 in 2021 [5]. Similarly, diabetes prevalence among younger populations has risen in the US. For example, the incidence of T2DM among adolescents aged 10-19 increased steadily from 9.0 per 100,000 in 2002 to 17.9 per 100,000 in 2018, reflecting an annual increase of 5.3% [6]. Regional analyses reveal that adolescents aged 15 to 19 in the Asia-Pacific region experienced an average annual percentage change (AAPC) of 2.97% and an incidence rate of 130.56 per 100,000. North Africa and the Middle East had the highest AAPC of 4.57% and an incidence of 123 per 100,000, while Europe showed the lowest growth rates with an AAPC of 0.45% [5,7].

Sex disparities in T2DM among adolescents and young adults show a complex pattern. Men have higher incidence rates, while women experience nearly four times higher mortality rates. Data from Saudi Arabia indicate an overall diabetes prevalence of 8.5% among individuals aged 15 years and above, with slightly higher rates in men (10.3%) than women (9.9%) [8,9]. According to the International Diabetes Federation (IDF), Saudi Arabia is among the countries most affected by early-onset T2DM, ranking seventh globally in overall diabetes prevalence. Approximately 5.3 million adults in the country are living with diabetes, representing a prevalence of 23.1%. The Saudi Pediatric and Youth Diabetes Registry reports that T2DM accounts for 6.4% of all pediatric diabetes cases, with an average age at diagnosis of just 9.08 years. These trends among youth are particularly concerning [10,11]. Multiple studies link the high incidence of diabetes in Saudi Arabia to epidemic levels, emphasizing the urgent need for rapid measures to address its effects [12].

Multiple studies attribute the growing prevalence of T2DM in Saudi Arabia and similar nations to high-calorie diets, limited physical activity, and genetic predisposition [13]. The Global Burden of Disease study identifies elevated body mass index (BMI) as the most significant risk factor for diabetes across all countries [5,14,15]. Urban environments that promote sedentary living, increased fast food consumption, and reduced physical activity have accelerated the onset of metabolic disorders among youth [13]. The implications are far-reaching. Individuals who develop T2DM in their teens or twenties face decades of chronic disease, with markedly higher risks of early complications and reduced life expectancy. This translates into substantial social and economic costs, burdening healthcare systems worldwide [16]. Despite the magnitude of the problem, awareness, screening, and tailored management strategies for young individuals remain insufficient. Research by the Centers for Disease Control and Prevention projects that the number of individuals under 20 years old with T2DM could reach approximately 220,000 by 2060, a striking 700% increase [17]. This review provides an in-depth overview of early-onset type 2 diabetes, focusing on its epidemiology, risk factors, pathophysiology, clinical presentation, and management.

## Review

Pathophysiology and risk factors

T2DM is a chronic metabolic disorder characterized by elevated blood glucose levels due to impaired insulin action and secretion. Understanding the underlying biological mechanisms and key risk factors is essential for effective prevention and management. Diet plays a pivotal role in T2DM risk. Evidence from the Nurses' Health Study demonstrated that overall diet quality strongly impacts diabetes risk [18]. Diets high in trans fats and with a high glycemic load were linked to increased diabetes risk, while higher intake of cereal fiber and polyunsaturated fats was associated with lower risk. Beyond weight-related factors, these findings emphasize the crucial role dietary choices play in diabetes prevention [18]. Physical inactivity is another major contributor to T2DM development. Research shows that sedentary lifestyles are closely associated with metabolic disorders. A meta-analysis revealed that each additional two hours of daily television viewing increases diabetes risk by approximately 14%. In contrast, spending an extra two hours per day standing or engaging in light physical activity reduces risk by about 12% [19].

Family history is a well-established independent risk factor for T2DM. It is frequently included in diabetes risk assessment and screening tools, typically as a binary variable. However, recent evidence shows that the age at which a family member is diagnosed with diabetes also significantly affects an individual's risk. A large Danish national cohort study followed two million individuals for a median of 14 years (24 million person-years) and identified 76,633 new cases of T2DM. The study found that individuals whose family members were diagnosed at younger ages had substantially higher diabetes risk themselves. For example, the incidence rate ratio (IRR) was 3.9 when a parent was diagnosed at age 50, compared with 1.4 at age 80. Similarly, the IRR was 3.3 when a sibling was diagnosed at age 30, compared with 2.0 at age 60 [20]. These findings highlight the importance of considering both genetic predisposition and shared environmental and lifestyle factors within families. Recording the age at diagnosis in family history provides valuable, easily obtainable information that can improve identification of high-risk individuals. These individuals may benefit from early monitoring, lifestyle counseling, and family-based preventive interventions [20].

Clinical presentation

Children diagnosed with T2DM may or may not present with symptoms. Common signs of hyperglycemia include polyuria, polydipsia, and unintentional weight loss-symptoms that can also indicate type 1 diabetes. Diabetic ketoacidosis (DKA) may be present at initial diagnosis for both types [21]. Detailed history from the patient presenting with DKA can aid clinicians in distinguishing between type 1 and early-onset type 2 DM. Type 1 diabetes typically manifests abruptly and is frequently accompanied by gastrointestinal symptoms such as vomiting and abdominal discomfort. In contrast, features more suggestive of type 2 diabetes include older age, higher BMI, a more gradual progression of hyperglycemic symptoms, elevated blood pressure, and increased triglyceride levels [22]. Despite these clinical cues, the most reliable method for differentiating between the two types remains the assessment of autoimmune biomarkers-particularly anti-GAD and islet-specific autoantibodies [23].

Screening for T2DM is recommended for high-risk children. The American Diabetes Association advises testing all overweight children with at least two additional risk factors, beginning at age 10 or at puberty onset, and repeating every two years [24]. Risk factors include a family history of T2DM in first- or second-degree relatives, maternal gestational diabetes, membership in certain ethnic groups (Native American, African American, Hispanic, or Asian/Pacific Islander), and signs of insulin resistance such as acanthosis nigricans, hypertension, dyslipidemia, or polycystic ovary syndrome [24]. Fasting blood glucose is the preferred screening method, though some guidelines now support HbA1c. When results are borderline or the child is asymptomatic, an oral glucose tolerance test can confirm the diagnosis. This test is unnecessary if the child displays classic diabetes symptoms or if fasting plasma glucose or HbA1c levels are significantly elevated on two separate occasions.

The American Diabetes Association (ADA) uses the same criteria for detecting diabetes in adolescents as for adults. Without hyperglycemic symptoms, four distinct methods can diagnose diabetes, each confirmed on a separate day. Fasting plasma glucose (FPG) greater than 7.0 mmol/L is the first method. The second involves plasma glucose of 11.1 mmol/L or higher two hours after an oral glucose tolerance test (OGTT), though it's important to note that the OGTT shows low reproducibility in adolescents, with less than 30% concordance between tests conducted several weeks apart. The third method is random plasma glucose levels of 11.1 mmol/L or higher when diabetic symptoms are present. Without such symptoms, hyperglycemia identified accidentally or during stressful situations (e.g., acute infection or surgery) may be temporary and should not be considered diagnostic of diabetes. In such cases, a repeat examination later will help confirm the diagnosis. The fourth method is glycated hemoglobin (A1C) of 48 mmol/mol (6.5%) or higher. The A1C criterion remains controversial because some studies show that individuals identified by this test only partially overlap with those identified by FPG or OGTT. For accuracy, A1C must be measured in a laboratory certified by the National Glycohemoglobin Standardization Program, rather than with a point-of-care device [25].

Complications and prognosis

The global prevalence of T2DM is rising sharply, driven by increasing obesity rates. Traditionally considered a disease of middle-aged and older adults, T2DM is now being diagnosed with growing frequency in younger populations. Individuals diagnosed at younger ages face higher risks of vascular complications, likely due to longer disease duration and a more adverse cardiovascular risk profile [26-28]. A systematic review and meta-analysis revealed an inverse relationship between age at diagnosis and complication risk, independent of current age. Each additional year in age at diagnosis was associated with a 4% lower risk of all-cause mortality, a 3% lower risk of macrovascular disease, and a 5% lower risk of microvascular disease [29]. Youth-onset T2DM leads to early development of serious complications in midlife, including neuropathy, retinopathy, diabetic kidney disease, and cardiovascular disease. The rapid and aggressive progression of T2DM in younger individuals highlights the urgent need for increased awareness, better understanding of underlying mechanisms, and development of effective interventions to reduce its impact [30].

Management and continuity of care

Lifestyle Management

Lifestyle modification remains the foundation of preventing and managing early-onset T2DM. Rising obesity rates and sedentary behaviors are key contributors to this growing problem [31]. Nutritional counseling plays a vital role, emphasizing the consumption of whole grains, vegetables, and lean meats while avoiding processed foods and sugary beverages. Behavioral counseling, combined with family support, is essential to help young individuals sustain these changes over time [32]. When lifestyle modifications and dietary changes fail to control blood sugar, medication becomes necessary. Metformin is the most commonly prescribed, improving insulin sensitivity and enhancing glucose uptake in muscle and fat tissues. If blood sugar remains uncontrolled, additional medications such as GLP-1 receptor agonists like liraglutide may be prescribed. In cases of severe hyperglycemia, insulin therapy may be required. Tailoring treatment to the individual and ensuring regular follow-up are essential to monitor progress and adjust therapy as needed [33].

Continuity of care is essential for adolescents with T2DM, especially during the transition from pediatric to adult health services. When responsibility for care shifts, gaps in follow-up often occur, leading to poor glycemic control and increased risk of complications [34]. Maintaining consistent contact with healthcare providers during this transition supports stable blood glucose levels and helps young people develop lifelong healthy habits. Family pediatricians play an important role in guiding children and adolescents on how to manage diabetes and understand the importance of regular medical visits. However, challenges frequently arise when patients move to adult care without a structured transition plan [34]. Family physicians are key to ensuring ongoing management. They provide continuous preventive care that addresses not only physical health but also emotional and social well-being. Sustained relationships with the same physician are linked to better control of blood glucose, cholesterol, and blood pressure, as well as improved adherence to medications and recommended screenings [35]. The most effective models of care rely on multidisciplinary teams-physicians, nurses, health educators, and counselors working together to address both medical and psychosocial needs [36]. Transition programs that coordinate communication between pediatric and adult clinics reduce the risk of patients being lost to follow-up and improve long-term outcomes [37]. Systems that include family doctor registration and long-term follow-up strengthen patient-provider relationships, while family and peer involvement further supports engagement in diabetes self-management [36].

Knowledge gaps and future directions

Early-onset type 2 diabetes is a growing health concern that differs from adult-onset T2DM in several important ways. The disease progresses more rapidly in young individuals, with earlier complications and poorer response to standard treatments [38]. However, the underlying reasons for these differences remain incompletely understood. Research indicates that puberty, obesity, and social determinants of health contribute to disease development and progression, but limited data links these factors comprehensively [38]. Treatment strategies also face substantial evidence gaps. The TODAY study showed that metformin alone is often insufficient for youth with T2DM, and even combination therapies do not always achieve optimal outcomes [39]. Newer agents, including GLP-1 receptor agonists and SGLT2 inhibitors, show promise, but additional studies are needed to evaluate their effectiveness and safety in younger populations under real-world conditions, not just controlled clinical trials [39]. From a clinical perspective, early and comprehensive screening with consistent follow-up is critical, yet many high-risk youth remain undiagnosed until complications arise. Health policy considerations are equally important; effective treatments will have limited impact if they are inaccessible or unaffordable for young patients [30,39].

## Conclusions

Early-onset T2DM represents one of the most pressing public health challenges of the 21st century. Its rising prevalence among adolescents and young adults reflects the consequences of modern lifestyles, physical inactivity, poor diet, and increasing obesity. Unlike adult-onset diabetes, the early-onset form progresses rapidly, with complications such as nephropathy, retinopathy, neuropathy, and cardiovascular disease often appearing before midlife. This reduces life expectancy and imposes substantial socioeconomic and healthcare burdens. Addressing this epidemic requires shifting focus from treatment to prevention: early screening of at-risk youth, healthier school and community environments, and family-centered lifestyle interventions.
